# Adjusting inspiratory rise time alters mechanical power in acute respiratory distress syndrome: opposing effects in pressure-controlled and volume-controlled ventilation modes

**DOI:** 10.3389/fmed.2026.1748263

**Published:** 2026-02-20

**Authors:** Furkan Tontu, Payam Rahimi, Yasemin Çelik, Esra Tontu, Zafer Çukurova, Sinan Aşar

**Affiliations:** 1 Department of Anesthesiology and Reanimation, Basaksehir Cam and Sakura City Hospital, Istanbul, Türkiye; 2 Department of Anesthesiology and Reanimation, Bakirkoy Dr. Sadi Konuk Training and Research Hospital, Istanbul, Türkiye; 3 Independent Software Developer (Sole Proprietorship), Antalya, Türkiye; 4 Department of Anesthesiology and Reanimation, Mardin Training and Research Hospital, Mardin, Türkiye

**Keywords:** acute respiratory distress syndrome, inspiratory rise time, geometric mechanical power, image processing, inspiratory-to-expiratory ratio, mechanical ventilators, Tslope, ventilator-induced lung injury

## Abstract

**Background:**

Mechanical power (MP), a predictor of ventilator-induced lung injury (VILI), is influenced by ventilatory parameters such as inspiratory rise time (Tslope). While Tslope affects the flow profile, its impact on MP in acute respiratory distress syndrome (ARDS) has not been thoroughly studied, particularly using the geometric method.

**Methods:**

In this prospective observational study, 30 deeply sedated and paralyzed ARDS patients were ventilated in both volume-controlled ventilation (VCV) and pressure-controlled ventilation (PCV) modes using a Maquet Servo-u ventilator. At inspiratory-to-expiratory (I:E) ratios of 1:2 and 1:1, Tslope was adjusted from 5 to 15%, and pressure–volume (P–V) loop screenshots were captured. Geometric mechanical power (MPtotal) was calculated based on the area enclosed by the P–V loops. A total of 720 images were analyzed.

**Results:**

In VCV mode, increasing Tslope from 5 to 15% led to a statistically significant increase in MPtotal: 0.8 J/min (5%) at I:E 1:2 and 0.1 J/min (1%) at I:E 1:1. Conversely, in PCV mode, Tslope prolongation resulted in a significant decrease in MPtotal: 1.8 J/min (12.5%) at I:E 1:2 and 1 J/min (7%) at I:E 1:1. No intrinsic PEEP was detected.

**Conclusion:**

Modifying Tslope alters MPtotal in opposing directions in PCV and VCV modes. In VCV, prolonging Tslope from 5 to 15% increased MP, whereas increasing the I:E ratio from 1:2 to 1:1 reduced MP. In PCV, prolongation of Tslope from 5 to 15% decreased MP by more than 1 J/min, and changes in the I:E ratio exerted minimal effects on MP.

## Graphical Abstract

**Figure:**
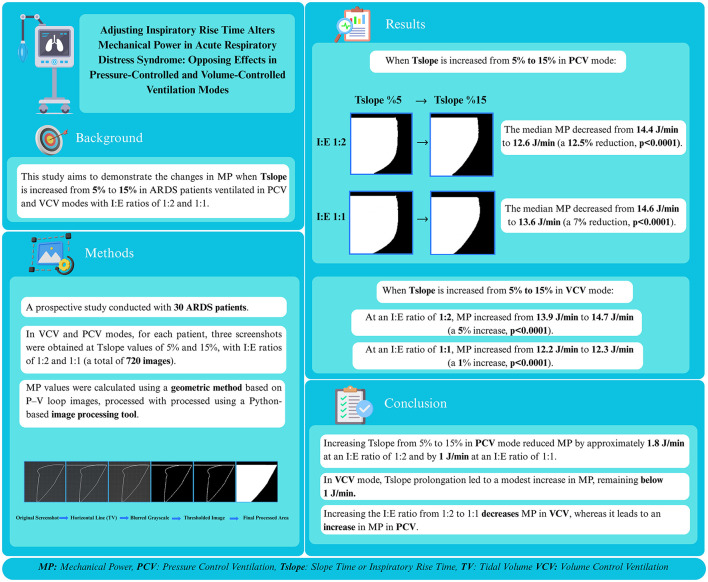

## Introduction

Mechanical power (MP) is a parameter calculated from respiratory mechanics to predict ventilator-induced lung injury (VILI) (1). MP is the energy transmitted by the mechanical ventilator to the lungs per min and is influenced by variables such as tidal volume (TV), airway resistance, compliance, and respiratory rate (1, 2). Therefore, changes in these parameters caused by inspiratory rise time (Tslope) are expected to affect MP (2). Especially in pressure-controlled ventilation (PCV) mode, the decremental flow pattern results in the highest energy transfer occurring during the early phase of inspiration (3). Prolonging Tslope slows the rise in inspiratory airway pressure (Pinsp), which may reduce tidal energy and, consequently, MP (3).

The flow profile and amplitude are thought to be associated with the development of VILI (4–6). MP has been widely studied, but prospective studies investigating the effect of Tslope, a parameter that influences the flow profile, on MP remain very limited (1, 7–13). In a study proposing a new equation for MP in PCV mode, Trinkle et al. (7) suggested that increasing Tslope may reduce MP, although they did not quantify the magnitude of this reduction. Later, Acicbe et al. (14) demonstrated this inverse relationship in acute respiratory distress syndrome (ARDS) patients using the MP equations (7, 8). However, their study did not employ the geometric method, which is considered the gold standard for MP measurement (1, 7, 14, 15). Furthermore, the effect of Tslope variation on MP has been largely overlooked in the context of volume-controlled ventilation (VCV) mode and remains unaddressed in the current literature. Therefore, the aim of this study was to investigate the effect of increasing Tslope on MP in ARDS patients ventilated in PCV and VCV modes at inspiratory-to-expiratory (I:E) ratios of 1:1 and 1:2.

## Materials and methods

This study was approved by the Clinical Research Ethics Committee of the University of Health Sciences Bakirköy Dr. Sadi Konuk Training and Research Hospital with decision number 2024/02-10. During ICU admission, the patients' relatives were thoroughly informed, and signed informed consent forms were obtained. This study was registered on ClinicalTrials.gov (NCT06413472).

### Study design and data collection

This study was conducted between 01.03.2024 and 01.06.2024 in the Intensive Care Unit of the Department of Anesthesiology and Reanimation at Bakirkoy Dr. Sadi Konuk Training and Research Hospital, a tertiary hospital in Istanbul. A total of 30 patients with ARDS, who were ventilated under deep sedation with VCV and PCV modes within 24–48 h of ICU admission, were included in the study. For the flow chart, see Supplementary Figure S1. Patients with chronic obstructive pulmonary disease (COPD), pregnant patients, patients with thoracopleural fistula, and hemodynamically unstable patients were excluded from the study.

### Ventilator settings

All patients were ventilated using the Maquet Servo-u (Gothenburg, Sweden) ventilator. Patient data were recorded on the ventilator at a scan speed of 20 mm/s. All patients were intubated with an 8.0 mm endotracheal tube. During the study, active humidification systems were not used, and passive humidification was provided with a heat and moisture exchanger (HME) filter. Positive end-expiratory pressure (PEEP) was set between 8–15 cmH_2_O and FiO_2_ between 40–80% to keep oxygen saturation (SpO_2_) between 88 and 92%.

In the Maquet Servo–U mechanical ventilator, inspiratory rise time (Tslope) is set by default at 5% and can be adjusted from 0 to 20%. In the present study, Tslope values of 5% and 15% were selected to represent the interquartile range (25th−75th percentiles) of the available adjustment spectrum. This approach was chosen to capture the majority of clinically relevant changes in inspiratory flow profile while avoiding extreme settings.

**Figure 1:**
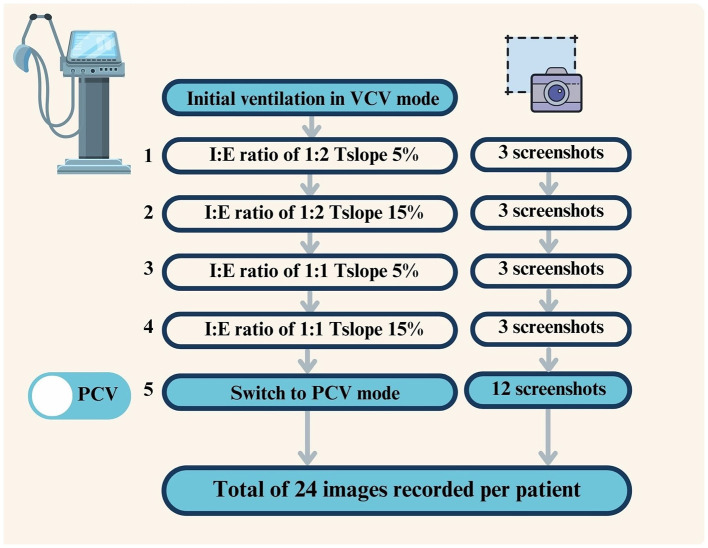
Study protocol for ventilator settings and screenshot acquisition in VCV and PCV modes (numbers indicate the sequential steps of the ventilatory protocol, corresponding to the order described in the Materials and methods Section).

Ventilator settings were modified according to a predefined stepwise protocol, as illustrated in Figure 1. (1) All patients were initially ventilated in VCV mode with an I:E ratio of 1:2 and Tslope set to 5%. After stabilization, three screenshots capturing complete pressure–volume (P–V) loops were recorded using the ventilator's screenshot function. (2) Tslope was then increased to 15% without any other changes in ventilator settings, and three additional P–V loop images were recorded, resulting in six images for VCV at an I:E ratio of 1:2. (3) The I:E ratio was subsequently adjusted to 1:1, and with Tslope set to 5%, three additional P–V loop images were recorded. (4) Tslope was then increased to 15% at an I:E ratio of 1:1, and three further images were obtained, yielding a total of 12 images in VCV mode. The ventilator was then switched to pressure-controlled ventilation (PCV). During the transition from VCV to PCV, the Servo-U ventilator automatically maintained the PEEP level and adjusted the inspiratory pressure above PEEP (ΔPinsp) to achieve the tidal volume previously delivered in VCV mode. (5) The identical sequence of I:E ratios and Tslope settings applied in VCV mode was repeated in PCV mode, resulting in 12 additional images. Overall, a total of 24 P–V loop images were recorded per patient (12 in VCV and 12 in PCV mode). Since 30 patients were included in the study, a total of 720 images were obtained. All screenshots were saved to an external USB storage device using the ventilator's data export function.

The presence of auto-PEEP was assessed by performing an expiratory hold maneuver after each Tslope adjustment to measure total PEEP (16). If the end-expiratory flow (Flowee) was close to zero, the absence of intrinsic PEEP was assumed (16). In PCV mode, Flowee values obtained at Tslope 5% and 15% with an I:E ratio of 1:2 were close to zero (mean ± SD: 0.014 ± 0.01 vs. 0.013 ± 0.01 L/s). Flowee data for Tslope 5% and 15% at an I:E ratio of 1:1 and 1:2 (mean ± SD and *P*-values) are provided in Supplementary Table S1. In VCV mode, end-expiratory flow was zero under all conditions; therefore, set PEEP was considered equivalent to total PEEP. Additionally, measured total PEEP values did not differ significantly from set PEEP values in either ventilation mode across all Tslope and I:E settings, indicating the absence of intrinsic PEEP in both PCV and VCV modes (*P* > 0.05 for all, Supplementary Table S2).

All respiratory mechanics throughout the study were recorded in the ImdSoft-Metavision/QlinICU Clinical Decision Support System. Mechanical ventilation parameters remained stable, with no adverse events necessitating adjustment. Hemodynamic parameters were continuously monitored at the bedside.

### Diagnosis and severity classification of ARDS

The diagnosis of ARDS was established according to the Berlin definition, based on clinical criteria, arterial blood gas analysis, and thoracic computed tomography findings (17). ARDS severity was classified using the PaO_2_/FiO_2_ ratio with PEEP ≥5 cmH_2_O as mild (200–300 mmHg), moderate (100–200 mmHg), or severe (< 100 mmHg) (17).

### Baseline patient characteristics and respiratory mechanics

Baseline demographic characteristics, disease severity scores, gas-exchange variables, and respiratory mechanics were recorded at ICU admission before protocolized ventilator adjustments. Sequential Organ Failure Assessment (SOFA), Acute Physiology and Chronic Health Evaluation II (APACHE II), age, gender, height, predicted body weight (PBW), ideal weight, and body mass index (BMI) parameters were extracted from the database using SQL queries and transferred to Excel. Respiratory parameters, including peak inspiratory pressure (Ppeak), PEEP, mean airway pressure (Pmean, automatically calculated by the ventilator), driving pressure (DP), respiratory rate (RR), tidal volume (TV), inspiratory time (Tinsp), static and dynamic compliance [Cstat and Cdyn, automatically calculated by the ventilator: Cstat = TV/DP, Cdyn = TV/(Ppeak – PEEP)], I:E ratio, end-tidal carbon dioxide (etCO_2_), SpO_2_, and initial arterial blood gas values at ICU admission [pH, PaO_2_, bicarbonate (${\text{HCO}}_{3}^{-}$), PaCO_2_, standard base excess (SBE)], were also retrieved from the database using SQL queries.

Statistical analyses were performed after calculating patient averages using Excel.

### Calculation of per-cycle energy and mechanical power

In VCV and PCV modes, inspiratory work (Wi) and its per-equivalent, MP, are most accurately calculated using the geometric method based on the P–V loop (1, 7, 11, 12). Millimetric-resolution P–V loop graphs generated by Servo-u mechanical ventilators allow for precise geometric calculation of the enclosed area from captured screen images. These loops begin at the PEEP level on the x-axis. Since PEEP remained constant throughout the respiratory cycle, the volume generated by PEEP was considered constant and not included in the variable portion of the volume axis, which starts from zero on the y axis (18).

**Figure 2:**
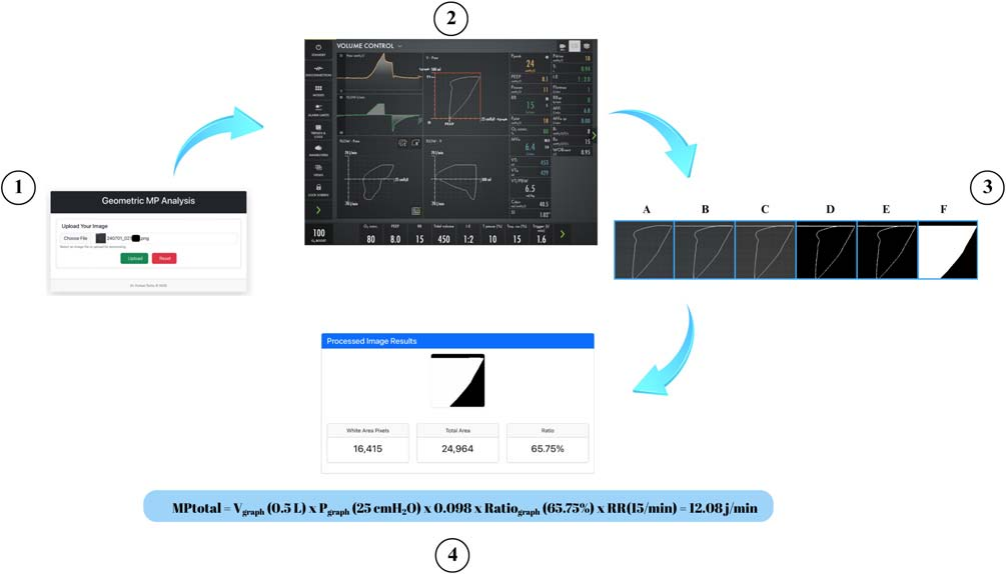
Image processing steps used for geometric measurement of the pressure–volume (P–V) loop. (1) Uploading the raw screenshot containing the P–V loop into the image processing tool; (2) Identifying the P–V loop area coordinates from the ventilator screenshot; (3A) Original cropped image of the P–V loop area; (3B) Drawing a horizontal line at tidal volume (VT) from Ppeak to 0 to correctly close the inspiratory loop area; (3C) Conversion of the adjusted image to grayscale; (3D) Application of binary thresholding to highlight the P–V loop contour; (3E) Morphological closing and contour extraction to isolate the loop boundaries; (3F) Flood fill algorithm to generate the enclosed white area representing inspiratory work; (4) Generating the processed image results, including white pixel area, total area, and the calculated ratio used for geometric mechanical power calculation, together with MPtotal calculation in a representative patient.

All images were analyzed in a randomized order to minimize observer bias. OpenCV, NumPy, and PIL libraries in Python were used to calculate the area enclosed by the P–V loops in the screenshots. First, the coordinates of the graph area within the screenshot were identified. To ensure accurate calculation of the enclosed area, a horizontal line at Volume = TV was drawn from Ppeak to 0 to correctly close the inspiratory loop area. The image was then converted to grayscale, followed by thresholding and morphological closing operations. Using the flood fill algorithm, foreground objects were isolated, and the ratio of white pixels (corresponding to the inspiratory work area) to the total area was calculated (18) (see Figure 2).

**Ratio**_**graph**_ = White area pixels/Total area

The total energy (E_total_area_) representing the total area was calculated by multiplying the tidal volume value (V_graph_) in liters on the y-axis by the pressure value (P_graph_) in kPa on the x-axis. 0.098 is the conversion factor from cmH_2_O to kPa (18).

**E**_**total_area**_ **(J)** **=** V_graph_ (L) X P_graph_ (cmH_2_O) X 0.098

The inspiratory work (Wi), representing the area enclosed by the P–V loop, was obtained by multiplying E_total_area_ by the Ratio_graph_ (18).

**Wi** = E_total_area_ x Ratio_graph_

As all patients were under deep sedation and fully paralyzed with rocuronium in controlled ventilation, Wi was used as a surrogate for each breath. The total mechanical power per minute (MPtotal) is calculated as follows (1, 18):

**MPtotal (J/min)** = RR (1/min) × Wi (J)

After transferring all respiratory mechanics data to Excel, the records were separated by mode (VCV or PCV), I:E ratio (1:2 or 1:1), and Tslope setting (5% or 15%), resulting in eight comparison groups:

VCV I:E 1:2 Tslope 5% and 15%,

VCV I:E 1:1 Tslope 5% and 15%,

PCV I:E 1:2 Tslope 5% and 15%,

PCV I:E 1:1 Tslope 5% and 15%.

### Statistical analysis

Patient characteristics were analyzed using descriptive statistics. Respiratory mechanics in PCV and VCV modes were compared using the Mann–Whitney *U* test. For each mode (PCV and VCV) and each I:E ratio (1:2 and 1:1), the repeated sequential measurements of Tslope 5% and 15% parameters were compared in pairs using the Wilcoxon test. In addition, measured PEEP values were compared with the corresponding set PEEP values using the Wilcoxon signed-rank test to assess the presence of intrinsic PEEP. In the statistical presentation, median [interquartile range (IQR)] and mean ± standard deviation (SD) values were used. Values with *P* < 0.05 were considered statistically significant. All statistical analyses were performed using GraphPad Prism version 10.4.1.

Statistical analyses were performed based on patient averages. Subgroup analyses were performed based on I:E ratios and Tslope values. A preliminary study was conducted with five patients to determine the required sample size. The primary outcome of the study was defined as the difference in MPtotal between Tslope 5% and 15% in PCV mode with an I:E ratio of 1:2. In PCV mode at an I:E ratio of 1:2, the mean ± SD MPtotal values for Tslope 5% and 15% were 14.3 ± 3 and 12.8 ± 3 J/min, respectively. A power analysis (G^*^Power version 3) with a 0.05 alpha error and 80% power indicated that 28 patients were required.

## Results

Baseline patient characteristics, gas-exchange variables, and respiratory mechanics at ICU admission are summarized in Table 1. The study included 30 patients with ARDS, of whom 4 (13%) had mild, 18 (60%) moderate, and 8 (27%) severe disease according to the Berlin definition (17). The mean age was 57.7 ± 15.5 years, and 30% were female. The mean APACHE II and SOFA scores were 26 ± 7 and 11 ± 4, respectively. ICU mortality was 50%, and the average duration of mechanical ventilation was 15 ± 9.3 days (Table 1).

**Table 1:** Baseline patient characteristics, gas-exchange variables, and respiratory mechanics at ICU admission.

**(*n*:30)**	**Mean ± SD**
Gender, female (%)	9 (30%)
Height, cm	172 ± 8.1
Weight, kg	91.9 ± 29.3
Body mass index, kg/m^2^	31.2 ± 9.3
Predicted body weight, kg	66 ± 8.8
Age, years	57.7 ± 15.5
pH	7.36 ± 0.08
PaCO_2_, mmHg	45 ± 17
PaO_2_, mmHg	91 ± 22
FiO_2_, %	62 ± 6
PaO_2_/FiO_2_, mmHg	150 ± 45
HCO_3_, mEq/L	21± 3.8
SBE, mEq/L	−1.1 ± 5
Pinsp, cmH_2_O	15.9 ± 3.9
PEEP, cmH_2_O	8.8 ± 2.9
Pplat, cmH_2_O	24.7 ± 4.9
Minute volume, L/min	7.8 ± 4.2
Compliance, ml/cmH_2_O	32.8 ± 8.8
APACHE II score	26 ± 7
ICU mortality, *n* (%)	15 (50%)
SOFA score	11 ± 4
LOS in ICU, days	21 ± 13
IMV time, day	15 ± 9.3

Patients characteristics at ICU admission. Data are presented as mean ± standard deviation (SD) or n (%).

All respiratory parameters shown represent baseline values obtained prior to protocolized changes in inspiratory rise time (Tslope) and I:E ratio. APACHE II, acute physiology and chronic health evaluation II; FiO_2_, fraction of inspired oxygen; ${\text{HCO}}_{3}^{-}$, bicarbonate; IMV, invasive mechanical ventilation; LOS, length of stay; MV, minute ventilation; PaCO_2_, partial pressure of carbon dioxide; PaO_2_, partial pressure of oxygen; PBW, predicted body weight; PEEP, positive end-expiratory pressure; Pinsp, peak inspiratory pressure; Pplat, plateau pressure; SBE, standard base excess; SOFA, sequential organ failure assessment.

Respiratory parameters at Tslope durations of 5% and 15% with I:E ratios of 1:1 and 1:2 in PCV and VCV modes are presented in detail in Table 2.

**Table 2:** Respiratory parameters measured at sequential Tslope (5% and 15%) with I:E ratios of 1:2 and 1:1 in PCV and VCV modes were analyzed using the Wilcoxon test based on patient mean values among 30 ARDS patients.

***n* = 30**	I:E 1:2 ratio			I:E 1:1 ratio		
	**Tslope 5%**	**Tslope 15%**	***p***	**Tslope 5%**	**Tslope 15%**	***p***
**PEEP, cmH**_2_ **O**						
VCV	8 (8–10)	8 (8–10)	>0.99	8 (8–10)	8 (8–10)	>0.99
PCV	8 (8–10)	8 (8–10)	>0.99	8 (8–10)	8 (8–10)	>0.99
**Pplat, cmH**_2_ **O**						
VCV	22.1 (21.8–24.4)	23 (21.5–24.4)	0.058	23 (22–24.6)	23 (22–24.8)	0.051
PCV	23.1 (21.7–25)	23.2 (21.8–25)	0.43	23.3 (21.8–25)	23 (21.8–24.8)	0.052
**DP, cmH**_2_ **O**						
VCV	14 (12–15)	14.1 (12– 15.4)	0.12	14.2 (12–15.5)	14.2 (12–15.8)	0.13
PCV	15 (13–16)	15 (13–16)	0.29	15 (13–16)	14.7 (13–16)	0.19
**RR,/min**						
VCV	15 (15–16)	15 (15–16)	0.75	15 (15–16)	15 (15–15.25)	>0.99
PCV	15 (15–15.3)	15 (15–15.7)	>0.99	15 (15–15.25)	15 (15–15.25)	>0.99
**Cstat, mL/cmH**_2_ **O**						
VCV	31.5 (25.8–37)	30.6 (26.3–37.4)	0.08	30.6 (25.5–37.1)	29.9 (25.5–36.6)	0.44
PCV	29.5 (24.9–32.4)	29.1 (24.1–31.8)	**< 0.0001**	31.5 (26–34.2)	31.6 (25.9–33.9)	0.44
**Cdyn, ml/cmH**_2_ **O**						
VCV	30.2 (25.9–35.2)	29.1 (25.8–35.2)	**< 0.0001**	30.6 (26–36.3)	29.9 (26.3–36.2)	0.25
PCV	30 (26.6–36.2)	30.3 (26–35.9)	0.21	30.4 (26.5–37.4)	30.7 (26.4–37.2)	0.15
**Ri, cmH**_2_**O** · **s/L**						
VCV	9.8 (7.4–13.8)	10.8 (7.9–15)	**< 0.0001**	8.3 (6.3–11.6)	8.6 (6.7–10.9)	0.76
**Pmean, cmH**_2_ **O**						
VCV	13.5 (12–14)	13 (12–14)	**0.001**	14.1 (13–15.3)	14 (13–15)	**0.004**
PCV	13.5 (12.3–14.5)	13 (12–14)	**< 0.0001**	16 (15–17)	15 (14.7–16)	**< 0.0001**
**Ppeak, cmH**_2_ **O**						
VCV	28.1 (26–31.1)	30.6 (28.2–33.7)	**< 0.0001**	25.4 (23.4–27.2)	25.7 (24–28.7)	**< 0.0001**
PCV	23.3 (22–25)	24 (22–25.1)	0.196	23.4 (22–25)	23.1 (22–25)	0.48
**TV, ml**						
VCV	443 (381–464)	442 (382–466)	0.91	446 (381–468)	444 (384–471)	0.26
PCV	433 (376–477)	414 (365–462)	**< 0.0001**	450 (394–493)	449 (393–491)	**0.035**
**Insp. flow** ^*^ **, L/min**						
VCV	33.5 (29.3–36)	43 (36.5–46)	**< 0.0001**	19 (17–20)	21 (18–22)	**0.0001**
PCV	51.5 (50–63.5)	38.5 (34–45.3)	**< 0.0001**	51 (46–54)	36 (32.5–38)	**0.0001**
**MPtotal, J/min**						
VCV	13.9 (12.5–15.9)	14.7 (12.7–16.9)	**< 0.0001**	12.2 (11.1–14)	12.3 (10.9–14.3)	**< 0.0001**
PCV	14.4 (12.7–17.3)	12.6 (11.6–15.3)	**< 0.0001**	14.6 (13.6–17.8)	13.6 (12.1–16.2)	**< 0.0001**

^*^Constant value in VCV, peak value in PCV.

Cdyn, dynamic compliance; Cstat, static compliance; DP, driving pressure; I:E, inspiratory-to-expiratory ratio; Insp., inspiratory; MPelastic, elastic mechanical power; MPtotal, geometrically calculated total mechanical power; PCV, pressure-controlled ventilation; PEEP, positive end-expiratory pressure; Pmean, mean airway pressure; Ppeak, peak airway pressure; Pplat, plato pressure; Ri, inspiratory resistance; RR, respiratory rate; TV, tidal volume; VCV, volume-controlled ventilation.

Values with *p* < 0.05 are indicated in bold.

At an I:E ratio of 1:2, prolongation of Tslope from 5 to 15% in VCV significantly decreased Pmean, while increasing Ppeak, inspiratory flow, and MPtotal [13.9 (12.5–15.9) vs. 14.7 (12.7–16.9) J/min, *P* < 0.0001]. In PCV, the same change in Tslope reduced Pmean, tidal volume, inspiratory flow, and MPtotal [14.4 (12.7–17.3) vs. 12.6 (11.6–15.3) J/min, *P* < 0.0001], whereas Ppeak remained stable (Table 2).

At an I:E ratio of 1:1, prolongation of Tslope from 5 to 15% in VCV, Ppeak and MPtotal increased [12.2 (11.1–14.0) vs. 12.3 (10.9–14.3) J/min, *P* < 0.0001], whereas in PCV, Pmean, inspiratory flow, and MPtotal decreased [14.6 (13.6–17.8) vs. 13.6 (12.1–16.2) J/min, *P* < 0.0001] (Table 2).

When the I:E ratio was increased from 1:2 to 1:1, MPtotal decreased in VCV (from 13.9 to 12.2 J/min at Tslope 5% and from 14.7 to 12.3 J/min at Tslope 15%), while the opposite effect was observed in PCV, where MPtotal increased (from 14.4 to 14.6 J/min at Tslope 5% and from 12.6 to 13.6 J/min at Tslope 15%) (Table 2).

PEEP, Pplat, DP, and RR did not show statistically significant changes when Tslope was increased from 5 to 15% in either VCV or PCV modes, at both I:E ratios of 1:1 and 1:2. In VCV mode, inspiratory resistance (Ri) increased significantly with Tslope prolongation at an I:E ratio of 1:2 [9.8 (7.4–13.8) vs. 10.8 (7.9–15) cmH_2_O·s/L, *p* < 0.0001], whereas at an I:E ratio of 1:1 the increase was not statistically significant [8.3 (6.3–11.6) vs. 8.6 (6.7–10.9) cmH_2_O·s/L, *P* = 0.76] (Table 2).

Pooled measurements obtained during the study protocol, rather than ICU baseline values, demonstrated that PEEP, Pmean, DP, RR, Cstat, Cdyn, and VTe values were comparable between PCV and VCV. Ppeak was significantly lower in PCV than in VCV (*P* < 0.0001), whereas inspiratory flow was significantly higher in PCV (*P* < 0.01). In contrast, MPtotal did not differ significantly between the two modes [13.8 (12.6–16.7) vs. 13.3 (11.9–15.3) J/min, *P* = 0.22] (Supplementary Table S3).

**Figure 3:**
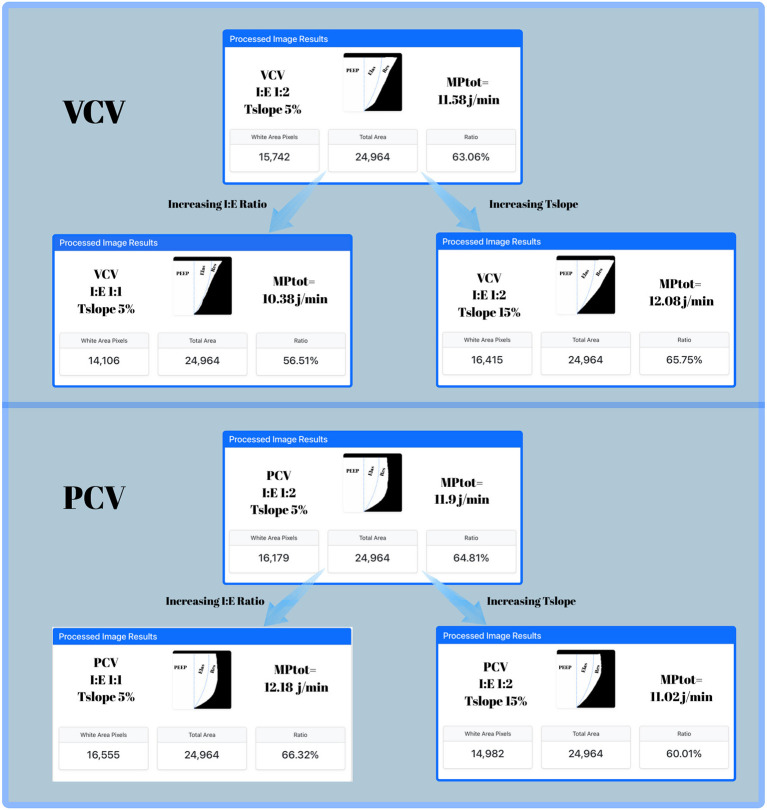
Dynamic pressure–volume (P–V) loops obtained from Servo-u ventilator screenshots in a patient passively ventilated in volume-controlled ventilation (VCV, upper panel) and pressure-controlled ventilation (PCV, lower panel) modes. The areas within the P–V loop representing the components of MP are illustrated. The PEEP area corresponds to the contribution of PEEP, Elas represents the elastic component of MP, and Res denotes the resistive component of MP. **Upper panel (VCV):** In VCV, prolongation of Tslope from 5 to 15% increased MPtotal (11.58 → 12.08 J/min), and resistive component of MP (MPres). Increasing the I:E ratio from 1:2 to 1:1 at Tslope 5% was associated with a decrease in MPtotal (11.58 → 10.38 J/min), and MPres. **Lower panel (PCV):** In PCV, by contrast, prolongation of Tslope from 5 to 15% reduced MPtotal (11.9 → 11.02 J/min), and MPres. Increasing the I:E ratio from 1:2 to 1:1 at Tslope 5% slightly increased MPtotal (11.9 → 12.18 J/min), and MPres.

Figure 3 shows, in a patient randomly selected from the study cohort, the effects of changes in Tslope and the I:E ratio on MP and its components—PEEP, elastic, and resistive.

## Discussion

MP is a clinically significant parameter associated with mortality in critically ill patients (19). This study employed the geometric method to quantify how modifying Tslope from 5 to 15% affects MP in both VCV and PCV modes at I:E ratios of 1:1 and 1:2.

In VCV mode, increasing Tslope from 5 to 15% at both I:E ratios of 1:2 and 1:1 was associated with an increase in MPtotal values. The median differences were calculated as 0.8 J/min (5%) for I:E 1:2, and 0.1 J/min (1%) for I:E 1:1, respectively. Although the increments in MP were below 1 J/min, they attained statistical significance. Notably, the increase in MP with Tslope prolongation is more pronounced at an I:E ratio of 1:2. To the best of our knowledge, no previous studies have investigated the effect of changes in Tslope duration on MP in VCV mode.

In PCV mode, increasing Tslope from 5 to 15% was associated with reductions in MPtotal values. The median differences were determined to be 1.8 J/min (12.5%) for I:E 1:2, and 1 J/min (7%) for I:E 1:1, respectively. At both I:E ratios, an increase in Tslope led to a reduction in MP exceeding 1 J/min. Trinkle et al. (7) were the first to propose that extending the Tslope duration in PCV mode results in a decrease in MP. Acicbe et al. demonstrated that in PCV-ventilated ARDS patients, increasing Tslope by 5% increments (from 5 to 20%), while keeping DP, RR, and PEEP constant, resulted in approximately a 1 J/min decrease in MP (14). This study experimentally validated the theoretical model proposed by Trinkle et al. (7), indicating that an extended Tslope in pressure-controlled ventilation decreases MP. Acicbe et al. supported this hypothesis through equation-based calculations in ARDS patients (14). This study demonstrated a similar inverse relationship between Tslope and MP in PCV using the gold standard geometric method.

High flow amplitude and flow profile have been identified as key determinants of VILI risk in ARDS patients (20–22). This study demonstrated that prolonging Tslope significantly reduced MPtotal in PCV mode, while it was associated with a modest increase in MPtotal in VCV mode. When examining the reasons for the observed increase in MP in VCV mode, a marked rise in inspiratory flow and a subtle increase in Ppeak, particularly at an I:E ratio of 1:2, were noted with increasing Tslope. In VCV, prolongation of Tslope increased inspiratory flow during a large portion of the breath, thereby elevating the resistive component of mechanical power (MPres). Similarly, in PCV mode, the reduction in MP with increasing Tslope appears to be primarily driven by a substantial decrease in inspiratory flow and a minimal reduction in tidal volume. In PCV, prolongation of Tslope from 5 to 15% produced a less aggressive flow profile during the early, high-flow phase of inspiration, which was associated with a reduction in MPres. Previous studies have shown that reducing inspiratory flow in PCV decreases per-cycle energy, whereas a 20% increase in flow in VCV results in a 37% increase in MP (1, 23). Comparable results have also been observed in experimental animal models of lung injury (24). Furthermore, an *in-silico* study in single- and multi-compartment lung models demonstrated that flow profile and amplitude—regulated by Tslope—are major determinants of strain and strain-related power distribution (25). Other studies have demonstrated that constant flow achieves a more balanced distribution of inspiratory energy and results in lower end-inspiratory energy delivery compared to the decelerating pattern observed in PCV (26, 27). Although reductions in inspiratory flow have been shown to decrease MP in several experimental and clinical models, this relationship may not be uniform across all physiological settings. In preterm lamb models, lowering inspiratory flow did not reduce MPtotal but was nevertheless associated with reduced epithelial injury, decreased permeability oedema, and improved lung morphology (28). These findings suggest that inspiratory flow characteristics may influence lung injury through mechanisms not fully captured by MPtotal, particularly in structurally immature lungs (28). The rapid delivery of per-cycle energy during early inspiration in ARDS patients has been debated as a potential contributor to lung injury, potentially disadvantaging the decelerating flow pattern in PCV mode (6). Accordingly, although prolongation of Tslope in PCV mode was associated with a reduction in MPtotal in the present study, this finding should be interpreted with caution. The decelerating flow pattern inherent to PCV may promote heterogeneous regional energy delivery and early inspiratory stress, such that a reduction in MPtotal does not necessarily imply a uniform reduction in regional lung stress, particularly in heterogeneous lungs such as ARDS (6, 29, 30).

Another parameter that influences gas flow in ventilation strategies is the I:E ratio (1). However, studies examining the relationship between the I:E ratio and MP in ARDS patients remain limited. In this study, the lowest MP values were observed at an I:E ratio of 1:1 in VCV mode and 1:2 in PCV mode. In VCV, increasing the I:E ratio reduced inspiratory flow throughout the entire breath, resulting in a decrease in MPtotal and MPres. In PCV, increasing the I:E ratio in PCV influenced only the late, low-flow portion of inspiration and therefore exerted negligible effects on MPtotal and MPres. Marini suggested that optimizing MP to mitigate the risk of VILI may involve favoring the constant flow pattern of VCV over the decelerating pattern of PCV, and recommended maintaining the I:E ratio between 1:1.5 and 1:1 (31).

This study demonstrates that changes in Tslope and I:E ratio primarily affect the MPres. The relative contribution of individual MP components to VILI and mortality, however, remains a matter of ongoing debate (32). Several experimental and clinical studies suggest that the dynamic elastic component of MP is more closely associated with adverse outcomes, including mortality, than the resistive component alone (29, 30). Experimental and physiological data indicate that resistive and PEEP-related energy components cannot be entirely discounted, as each may contribute to lung injury under specific ventilatory conditions when applied repetitively over time (32, 33). High MP levels—above approximately 12 J/min in ARDS and 17 J/min in non-ARDS—have been associated with significant lung injury in both experimental and clinical studies (19, 34, 35). Exceeding these thresholds is thought to promote progressive lung damage by reducing ventilated lung units (the “baby lung”), increasing stress concentration in remaining regions, and enhancing secondary injury mechanisms (32). In the present study, adjustments of the I:E ratio and Tslope in both ventilation modes resulted in reductions of up to approximately 2 J/min in MPtotal. Given the threshold-dependent nature of MP–related lung injury, even relatively modest changes in MP may be physiologically relevant when occurring near critical values (34). For example, increases in MP within lower ranges (e.g., 8 to 10 J/min) may have limited biological impact, whereas similar absolute increases at higher levels (e.g., 12 to 14 J/min) may be associated with a disproportionately greater risk of lung injury in ARDS patients. Nevertheless, the present study was not designed to evaluate clinical outcomes, and these findings should be interpreted as demonstrating association rather than causation.

MPtotal values were slightly lower in VCV compared to PCV; however, this difference was not statistically significant. In addition, Ppeak was higher and inspiratory flow was lower in VCV mode. These findings are consistent with literature (36).

MP, when calculated using the geometric method, is typically derived through numerical integration of the pressure–volume curve. This integration-based approach to geometric MP calculation is known to be a multi-step, time-consuming process that requires technical expertise (7). The present study represents the first clinical application of this newly validated geometric MP technique in patients, directly deriving per-cycle energy and mechanical power from ventilator screen pressure–volume loops via image-processing technology (18). With the rapid advancement of mechanical ventilator technology and artificial intelligence applications, complex calculations such as MP can soon be performed easily, quickly, and reliably at the bedside (18, 37, 38). These measurements can be derived directly from ventilator screen images, without the need for formula-based computation, thus supporting clinical decision-making.

### Limitations

This study has several limitations. First, the mechanical ventilator used (Maquet Servo-u) measures all respiratory mechanics from the proximal limb, which may introduce a margin of error compared to intratracheal measurements, potentially resulting in minor discrepancies in the findings. Second, the captured P–V loop images reflect only lung mechanics; assessing the chest wall's contribution to per-cycle energy and MP would require transpulmonary pressure measurements. Third, despite all screenshots being obtained at high resolution using the same ventilator model and standardized display settings, and image quality being verified by the investigators prior to analysis using a uniform Python-based platform, image-based MP calculation remains dependent on screenshot quality and device-specific display characteristics. Moreover, the absence of formal inter- and intra-observer variability assessment may limit generalizability. At present, the image-based geometric method does not allow detailed quantitative separation of all MP components, which limits component-level analysis in the current study. Fourth, inspiratory resistance (Ri) could not be measured in PCV mode, which limited our ability to evaluate this parameter. Additionally, although arterial blood gas values were recorded at ICU admission, follow-up measurements (e.g., PaO_2_, PaCO_2_) after adjustments to the I:E ratio or Tslope were not assessed. While the study primarily focused on the impact of Tslope modifications on MP, their effects on oxygenation parameters and clinical outcomes were not evaluated. Finally, reliance on a single ventilator platform limits generalizability; nevertheless, the underlying image-based geometric approach and flow-shaping principles are transferable to other modern ventilators with comparable capabilities.

## Conclusion

The inspiratory flow profile is a key determinant of mechanical power (MP). In VCV, prolonging Tslope from 5 to 15% increased MP, whereas increasing the I:E ratio from 1:2 to 1:1 reduced MP. In PCV, prolongation of Tslope from 5 to 15% decreased MP by up to 2 J/min, and changes in the I:E ratio exerted minimal effects on MP. These mode-specific responses underscore the importance of tailoring flow-shaping parameters when aiming to minimize MP; however, whether such reductions in MP translate into reduced VILI cannot be inferred from the present study.

## Data Availability

The raw data supporting the conclusions of this article will be made available by the authors upon reasonable request and subject to institutional approval, in accordance with ethical and legal requirements.
