# Supplementary material for: Adjusting inspiratory rise time alters mechanical power in acute respiratory distress syndrome: opposing effects in pressure-controlled and volume-controlled ventilation modes
Source: Front Med (Lausanne). 2026 Feb 20;13:1748263. doi: 10.3389/fmed.2026.1748263 (PMC12963225; doi:10.3389/fmed.2026.1748263)
Supplement: Supplementary file 1 [file Data_Sheet_1.docx]

**Adjusting Inspiratory Rise Time Alters Mechanical Power in Acute Respiratory Distress Syndrome: Opposing Effects in Pressure-Controlled and Volume-Controlled Ventilation Modes**

Furkan Tontu, Payam Rahimi, Yasemin Çelik, Esra Tontu, Zafer Cukurova, Sinan Asar

**Suplemantary Data**

For the flow chart, see **Figure 1S**.


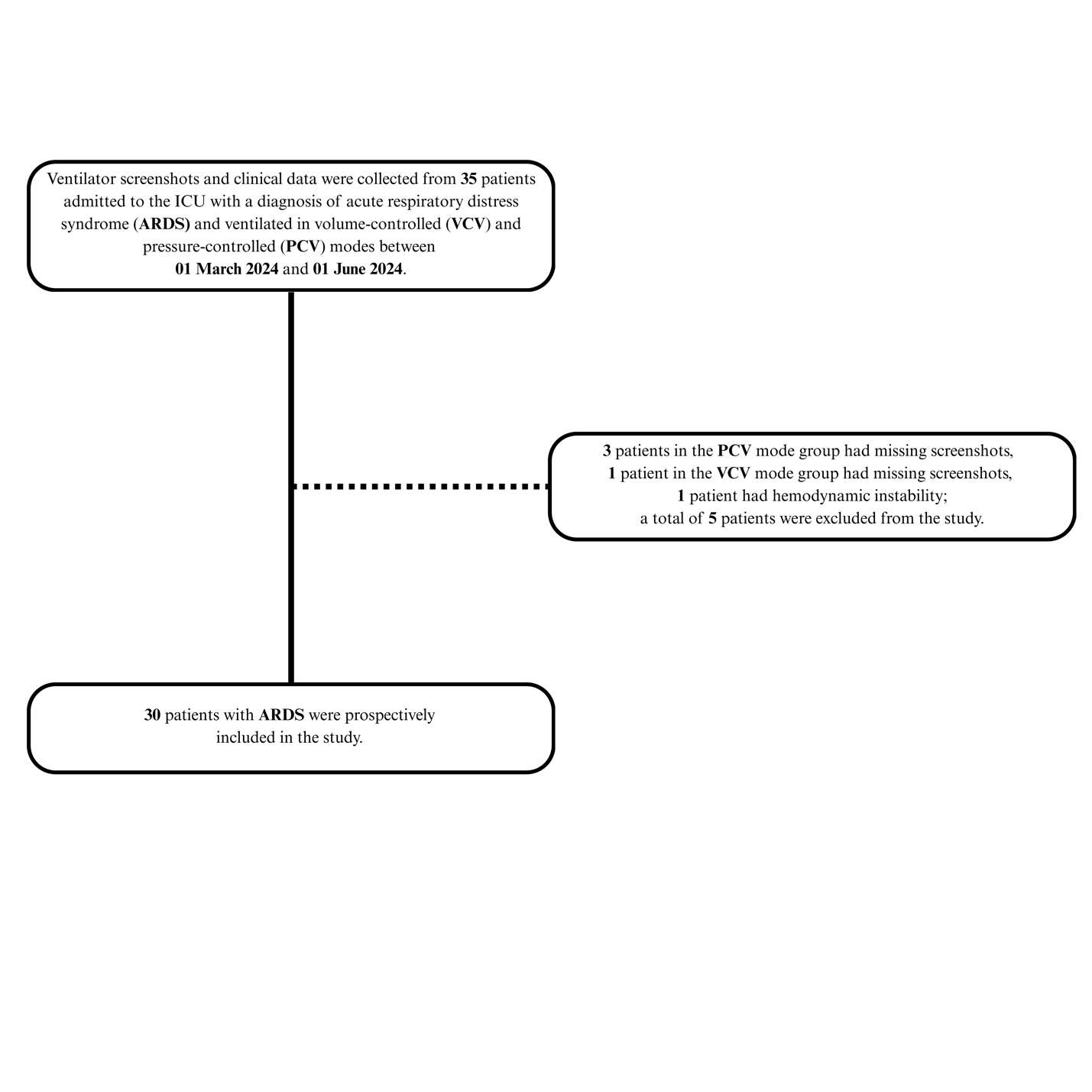


# **Figure 1S. Flow chart of patients**

Flowee measurements (mean ± SD, L/s) in PCV mode at Tslope values of 5% and 15% for I:E ratios of 1:2 and 1:1. No significant difference was observed at an I:E ratio of 1:1 (p = 0.4), while a statistically significant reduction was detected at an I:E ratio of 1:2 (p = 0.0006) (**Table 1S**).

**Table 1S. Mean ± SD values of expiratory Flowee (L/s) in pressure-controlled ventilation mode at Tslope settings of 5% and 15% with I:E ratios of 1:2 and 1:1.**

|  | **I:E 1:2 Ratio** | | | **I:E 1:1 Ratio** | | |
| --- | --- | --- | --- | --- | --- | --- |
|  | **Tslope %5** | **Tslope %15** | **p** | **Tslope %5** | **Tslope %15** | **p** |
| **Flowee, L/sn** |  | | |  | | |
| PCV | 0.014 ± 0.01 | 0.013 ± 0.01 | **0.0006** | 0.034 ± 0.02 | 0.035 ± 0.02 | 0.4 |

Flowee, end-expiratory flow; I:E, inspiratory-to-expiratory ratio; PCV, pressure-controlled ventilation; SD, standard deviation; Tslope, inspiratory rise time.

Values with p < 0.05 are indicated in **bold**.

The results of paired comparisons between set and measured total PEEP across all ventilation modes, I:E ratios, and Tslope settings are summarized in **Table 2S**.

**Table 2S.** **Comparison of set and measured total PEEP values across ventilation modes, I:E ratios, and Tslope settings.**

| **Mode** | **I:E** | **Tslope** | **Set PEEP** | **Measured PEEP** | **p** |
| --- | --- | --- | --- | --- | --- |
| **PCV** | **1:2** | **5%** | 8 (8-10) | 8.05 (7.92-9.97) | 0.29 |
|  |  | **15%** | 8 (8-10) | 8.09 (7.92-9.96) | 0.15 |
|  | **1:1** | **5%** | 8 (8-10) | 8.15 (8-10) | 0.11 |
|  |  | **15%** | 8 (8-10) | 8.15 (7.94-10) | 0.23 |
| **VCV** | **1:2** | **5%** | 8 (8-10) | 8.05 (7.9-10) | 0.09 |
|  |  | **15%** | 8 (8-10) | 8.06 (7.96-10) | 0.8 |
|  | **1:1** | **5%** | 8 (8-10) | 8.15 (8-10) | 0.07 |
|  |  | **15%** | 8 (8-10) | 8.19 (8.02-10) | 0.13 |

**Table 3S. Comparative respiratory mechanics during PCV and VCV modes across the study protocol (Mann–Whitney U Test)**

|  | **PCV** | **VCV** | **p** |
| --- | --- | --- | --- |
| **PEEP** | 8 (8-10) | 8 (8-10) | 0.93 |
| **Pmean** | 14.4 (13.6-15.4) | 13.8 (12.5-14.7) | 0.11 |
| **Ppeak** | 23(22-25) | 27(26-30) | **<0.0001** |
| **DP** | 14.8 (13-16) | 14.2 (12-15.3) | 0.25 |
| **RR** | 15 (15-15.3) | 15 (15-16) | 0.83 |
| **Cstat** | 30.8 (25.3-32.7) | 30.6 (25.5-37) | 0.58 |
| **Cdyn** | 30.3 (26.5-36.9) | 29.8 (26-35.7) | 0.84 |
| **TV** | 430 (387-479) | 444 (382-467) | 0.86 |
| **Inspiratory Flow*** | 45 (42-54) | 38.3 (33-41) | **<0.01** |
| **MPtotal** | 13.8 (12.6-16.7) | 13.3 (11.9-15.3) | 0.22 |

* Constant value in VCV, peak value in PCV

**Values represent pooled measurements obtained during the study protocol and are not baseline measurements.** Cdyn, dynamic compliance; Cstat, static compliance; DP, driving pressure; Energy, per-cycle mechanical energy; MPelastic, elastic component of mechanical power; MPtotal, total mechanical power calculated by geometric method; PCV, pressure-controlled ventilation; PEEP, positive end-expiratory pressure; Pmean, mean airway pressure; Ppeak, peak inspiratory pressure; RR, respiratory rate; TV, tidal volume; VCV, volume-controlled ventilation.

Values with p < 0.05 are indicated in **bold**.

As shown in **SDM** **Table 3S**, PEEP, Pmean, DP, RR, Cstat, Cdyn, and VTe values were comparable between PCV and VCV. Ppeak was significantly lower in PCV than in VCV (p < 0.0001), whereas inspiratory flow was significantly higher in PCV (p < 0.01). In contrast, MPtotal did not differ significantly between the two modes [13.8 (12.6–16.7) vs. 13.3 (11.9–15.3) J/min, p = 0.22].
